# Antimicrobial Efficacy of Un-Ionized Ammonia (NH_3_) against Salmonella Typhimurium in Buffered Solutions with Variable pH, NH_3_ Concentrations, and Urease-Producing Bacteria

**DOI:** 10.1128/spectrum.01850-21

**Published:** 2022-01-19

**Authors:** Alan Gutierrez, Arie H. Havelaar, Keith R. Schneider

**Affiliations:** a Food Science and Human Nutrition Department, University of Florida, Gainesville, Florida, USA; b Department of Animal Sciences, Emerging Pathogens Institute and Food Systems Institute, University of Florida, Gainesville, Florida, USA; Health Canada

**Keywords:** *Salmonella*, ammonia, antimicrobial, urease-producing bacteria, urease, modeling, poultry litter, antimicrobial activity

## Abstract

The presence of Salmonella in poultry litter, when used as a biological soil amendment, presents a risk for the preharvest contamination of fresh produce. Poultry litter is rich in organic nitrogen, and previous studies have suggested that ammonia (NH_3_) in poultry litter may affect the survival of Salmonella. Salmonella enterica serovar Typhimurium was inoculated into buffer solutions to characterize the pH dependency, minimum antimicrobial concentration, and efficacy of NH_3_ production. In solutions with 0.4 M total ammonia nitrogen (TAN) at various pH levels (5, 7, 8, and 9), significant inactivation of Salmonella only occurred at pH 9. Salmonella was reduced by ∼8 log CFU/mL within 12 to 18 h at 0.09, 0.18, 0.26, and 0.35 M NH_3_. The minimum antimicrobial concentration tested was 0.04 M NH_3_, resulting in an ∼7 log CFU/mL reduction after 24 h. Solutions with urea (1% and 2%) and urease enzymes rapidly produced NH_3_, which significantly reduced Salmonella within 12 h. The urease-producing bacterium Corynebacterium urealyticum showed no antagonistic effects against Salmonella in solution. Conversely, with 1% urea added, *C*. *urealyticum* rapidly produced NH_3_ in solution and significantly reduced Salmonella within 12 h. Salmonella inactivation data were nonlinear and fitted to Weibull models (Weibull, Weibull with tailing effects, and double Weibull) to describe their inactivation kinetics. These results suggest that high NH_3_ levels in poultry litter may reduce the risk of contamination in this biological soil amendment. This study will guide future research on the influence of ammonia on the survival and persistence of Salmonella in poultry litter.

**IMPORTANCE** Poultry litter is a widely used biological soil amendment in the production of fresh produce. However, poultry litter may contain human pathogens, such as Salmonella, which introduces the risk of preharvest produce contamination in agricultural fields. Ammonia in poultry litter, produced through bacterial degradation of urea, may be detrimental to the survival of Salmonella; however, these effects are not fully understood. This study utilized aqueous buffer solutions to demonstrate that the antimicrobial efficacy of ammonia against Salmonella is dependent on alkaline pH levels, where increasing concentrations of ammonia led to more rapid inactivation. Inactivation was also demonstrated in the presence of urea and urease or urease-producing Corynebacterium urealyticum. These findings suggest that high levels of ammonia in poultry litter may reduce the risk of contamination in biological soil amendments and will guide further studies on the survival and persistence of Salmonella in poultry litter.

## INTRODUCTION

Each year in the United States, Salmonella causes approximately 1 million illnesses, 19,000 hospitalizations, and 378 deaths (1, 2). Salmonellosis outbreaks are generally associated with the consumption of poultry, meat, eggs, and, increasingly, produce (3–5). Between 1998 and 2013, 972 outbreaks were attributed to the consumption of raw produce, and Salmonella was the most common bacterial cause of these outbreaks (4). From 2010 to 2017, 56 multistate outbreaks of salmonellosis were associated with fresh produce, causing a total of 3,778 illnesses (5). The poultry industry has long struggled to control Salmonella contamination among broiler and layer chickens (6). Poultry litter is a mixture of poultry excreta, feathers, wasted feed, and bedding materials from poultry houses (7). Poultry litter is recognized as an environmental source of Salmonella dissemination among birds in poultry houses (8–10). In the United States, an estimated 14 million tons of poultry litter are produced each year by poultry production operations, with much of it being land applied to fertilize agricultural soils (11, 12). The presence of Salmonella and other pathogenic microorganisms in poultry litter presents a risk for the preharvest contamination of fresh produce (7, 13). Investigating the factors that affect the survival of Salmonella in poultry litter may help mitigate these risks.

Poultry litter is desired as an organic fertilizer for its high nitrogen content (14). The predominant forms of organic nitrogen in litter, urea, and uric acid are rapidly broken down to ammonia by urease and uricase enzymes produced by microorganisms naturally present in the litter (15). In poultry litter, ammonia exists in equilibrium between two forms: NH_3_ (un-ionized ammonia) and NH_4_^+^ (ammonium). Total ammonia nitrogen (TAN) is the sum of both of these chemical species (16). The equilibrium between these two chemical species is dependent on pH and temperature, where increases in either one will drive the equilibrium toward NH_3_. At alkaline pH levels, NH_3_ becomes the predominant species in solutions (17, 18). NH_3_ is the more toxic form, and its toxicity toward humans, animals, and bacteria has been demonstrated by several studies (17, 19–21). The inactivation of bacteria by NH_3_ is caused by the depletion of cytoplasmic potassium ions (K^+^), which are lost as NH_3_ enters the cell, causing the uptake of protons to maintain the cytoplasmic pH and requiring K^+^ efflux to drive this proton uptake (22). Ammonia production in litter results in the loss of nitrogen through ammonia volatilization or off-gassing. Ammonia volatilization increases significantly when litter pH rises above 8, and poultry litter can lose over 50% of its nitrogen through ammonia volatilization (23–25). The build-up of ammonia gas in poultry houses has been shown to have numerous detrimental effects on the health of the birds, poultry farmworkers, and the environment (24, 26).

Several studies have suggested that ammonia in poultry litter may be partly responsible for reducing Salmonella populations in poultry litter (27–32). Turnbull and Snoeyenbos (28) reported that Salmonella enterica serovar Typhimurium (ST) in poultry litter was reduced by more than 8 log CFU/mL within 8 h when exposed to NH_3_ gas at a water activity (*a_w_*) of approximately 1.00. Another study showed that *S*. Typhimurium, Escherichia coli O157:H7, and Listeria monocytogenes could be reduced by 2.5 to 4.0 log CFU/g in poultry manure dried to 10% moisture and treated with 1% NH_3_ gas for 24 h (31). Mendonça et al. (33) used 2,411 ppm NH_3_ gas to successfully decontaminate poultry litter in broiler houses contaminated with *S*. Heidelberg. The antimicrobial effects of ammonia on Salmonella have also been studied in aqueous solutions (19, 34, 35). Ricke et al. (34) studied the inhibitory effects of ammonium salts on the growth of *S*. Typhimurium in tryptic soy broth (TSB) at alkaline pH levels. Comparing the ammonium salts by MIC, they found that the most and least inhibitory salts were NH_4_OH (50 mM) and (NH_4_)_2_SO_4_ (>600 mM), respectively (34). Koziel et al. (19) reported that 0.1 M NH_3_ reduced *S*. Typhimurium by 8 log CFU/mL within 24 h in phosphate-buffered saline (PBS) solutions (pH 9.0).

The antimicrobial effects of NH_3_ on Salmonella in poultry litter and aqueous solutions have been demonstrated in limited studies. The objective of this study was to characterize the interactions between Salmonella, NH_3_, and the mechanisms of ammonia production in litter (i.e., urea, urease, and urease-producing bacteria). This study utilized a simplified buffer system to isolate these interactions from confounding factors in litter, such as microflora and *a_w_*. These interactions were studied in three experiments that were designed to (i) examine the effects of pH on the antimicrobial efficacy of NH_3_, (ii) determine the minimum antimicrobial concentration of NH_3_, and (iii) examine the production and antimicrobial efficacy of NH_3_ using urease and urease-producing bacteria. Salmonella inactivation data from the NH_3_ concentration, urease, and urease-producing bacteria experiments were fitted to nonlinear survival models to describe the inactivation kinetics of each treatment.

## RESULTS

### pH treatments.

**FIG 1:**
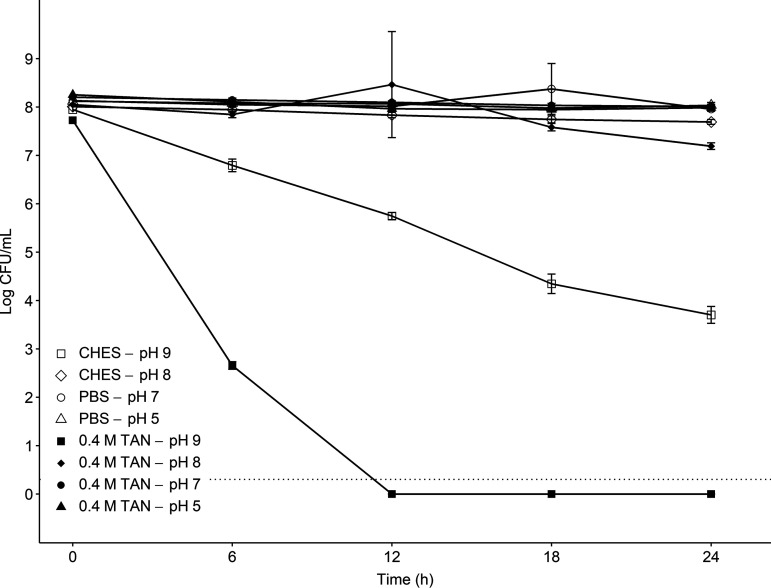
Survival of Salmonella Typhimurium in buffer solutions (PBS, CHES) at various pH levels (5, 7 to 9), with and without ammonium sulfate added (0.4 M TAN). Data points are presented as mean ± standard deviation log CFU/mL (*n* = 3). The dotted line indicates the LOD (0.30 log CFU/mL).

The survival of Salmonella in solutions at various pH levels (5, 7, 8, and 9) with and without ammonium sulfate added (0.4 M TAN) is shown in Fig. 1. The calculated un-ionized ammonia concentrations (M NH_3_) corresponding to each pH treatment with 0.4 M TAN added are reported in Table 1. The mean ST populations (log CFU/mL), pH, TAN (ppm), and un-ionized ammonia concentrations (M NH_3_) at each sampling time are reported in Tables S1, S5, S6, and S7 in the supplemental material, respectively. After 24 h, ST populations in all treatments under pH 9 remained above 7 log CFU/mL. Increases were observed in PBS (pH 7) at 18 h and 0.4 M TAN (pH 8) at 12 h, although these increases were not significant (*P* > 0.05) compared to the other treatments under pH 9. Significant reductions (*P* < 0.05) of 1.16 and 5.07 log CFU/mL were observed after 6 h in the *N*-cyclohexyl-2-aminoethanesulfonic acid (CHES; pH 9) and 0.4 M TAN (pH 9) treatments, respectively. After 12 h, ST populations in the 0.4 M TAN (pH 9) treatment fell below the limit of detection (LOD; 0.30 log CFU/mL). In the CHES (pH 9) treatment, ST populations were reduced to 3.70 log CFU/mL (4.25-log reduction) after 24 h (Fig. 1). Among the treatments with 0.4 M TAN added, the pH 9 CHES solution had the highest concentration of un-ionized ammonia. In these treatments, over 50% of the TAN was in the form of NH_3_ at pH 9 compared to less than 10% in solutions at a pH of ≤8 (Table 1).

**TABLE 1:** Calculated total ammonia nitrogen (TAN) and un-ionized ammonia (NH_3_) concentrations for pH experiment solutions with ammonium sulfate added

Buffer solution	pH	TAN (M)	TAN (ppm)	NH_3_ (M)^*a*^	NH_3_ (ppm)^*a*^
PBS	5.0	0.3964	5,552	3.18 × 10^−5^	0.541
PBS	7.0	0.3964	5,552	3.15 × 10^−3^	53.7
CHES	8.0	0.3964	5,552	2.94 × 10^−2^	501
CHES	9.0	0.3964	5,552	1.76 × 10^−1^	3,002

a Calculated using equations 1 and 2, at 30°C.

### Un-ionized ammonia treatments.

**FIG 2:**
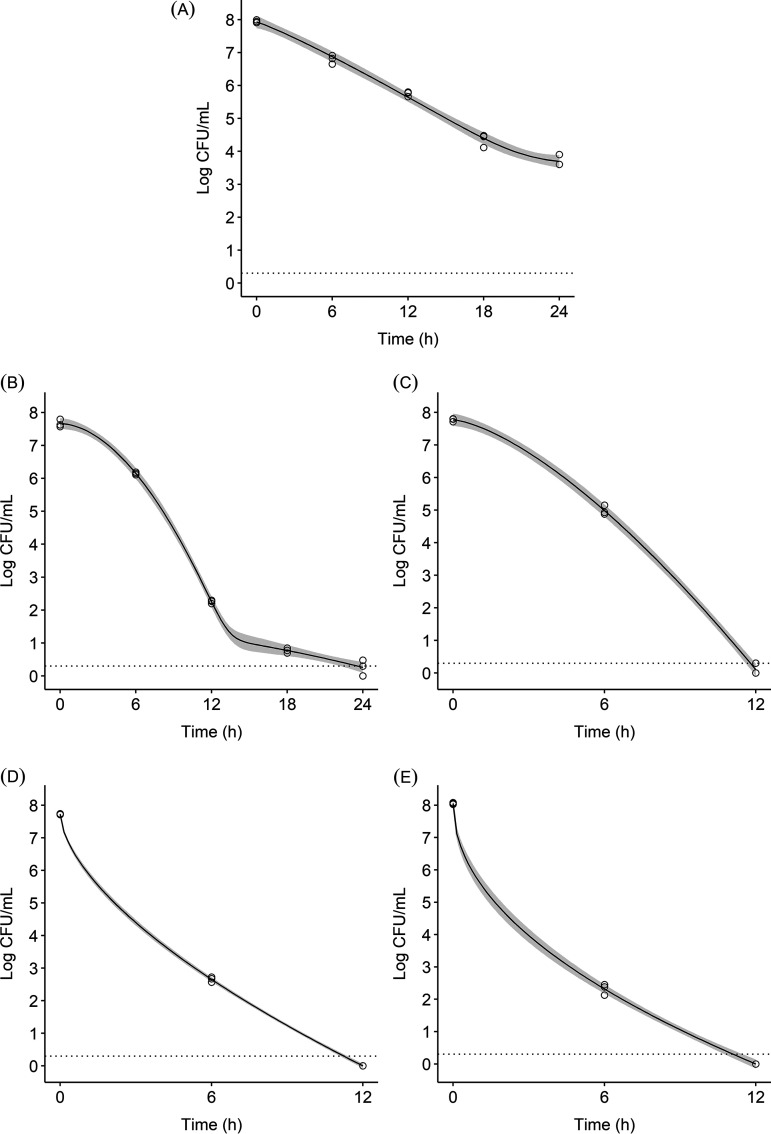
Salmonella Typhimurium survival in un-ionized ammonia treatments fitted by Weibull models for CHES, pH 9 (A), 0.04 M NH_3_ (B), 0.09 M NH_3_ (C), 0.18 M NH_3_ (D), and 0.26 M NH_3_ (E). Data points for each sampling time (*n* = 3), Weibull model curves, and their 95% confidence intervals (shaded area) are shown. The dotted line indicates the LOD (0.30 log CFU/mL).

Salmonella inactivation at various levels of un-ionized ammonia (M NH_3_) fitted to Weibull curves is shown in Fig. 2. The mean ST populations (log CFU/mL), pH, TAN (ppm), and un-ionized ammonia concentrations (M NH_3_) at each sampling time are reported in Tables S2, S5, S6, and S7, respectively. Salmonella populations in all treatments, except the control (CHES, pH 9; Fig. 2A), approached the LOD within 12 to 24 h. In the 0.04 M NH_3_ treatment, the ST concentration was reduced to 0.26 log CFU/mL (7.40-log reduction) after 24 h (Fig. 2B). The 0.09 M NH_3_ treatment approached the LOD at 12 h (Fig. 2C). The 0.18 and 0.26 M NH_3_ treatments were the quickest to fall below the LOD at 12 h (Fig. 2D and E, respectively). In the 0.35 M NH_3_ treatment, mean ST populations were reduced to 0.36 log CFU/mL (7.62-log reduction) at 6 h, followed by increased recovery (0.57-log increase) at 12 h. However, this increase was not significant (*P* > 0.05) compared to the other ammonia treatments of 0.09, 0.18, and 0.26 M NH_3_ at this sampling time. Salmonella in the 0.35 M NH_3_ treatment fell below the LOD at 18 h.

The parameter estimates for the fitted Weibull curves, their 95% confidence intervals, and goodness-of-fit measures (Akaike information criterion [AIC] and Bayesian information criterion [BIC]) describing ST survival in un-ionized ammonia solutions are presented in Table 2. The control treatment (CHES, pH 9) was fitted well to three models with tailing effects: bilinear (AIC: −54.44; BIC: −52.31), Weibull (AIC: −53.32; BIC: −50.49), and Geeraerd (AIC: −52.98; BIC: −50.86). With AIC and BIC values within two points for these models (36), the Weibull model with tailing effects was selected to be comparable with model fits for the other treatments. The resulting Weibull curve was slightly concave (*P* = 1.12) with a tailing effect (*N*_res_) starting at 3.62 log CFU/mL (Fig. 2A). The double Weibull model represented the best fit for the 0.04 M NH_3_ treatment. This survival curve was concave (*P* = 1.85) until it reached an inflection point after the 12-h sampling point (Fig. 2B). The 0.09 M NH_3_ treatment was best fitted to the Weibull model and the bilinear and Geeraerd models with shoulder effects, where all three models had the same AIC and BIC values of −33.97 and −33.38, respectively. The Weibull model was selected as the preferred model. The 0.18 and 0.26 M NH_3_ treatments were best fitted to Weibull survival curves, with no shoulder or tailing phenomena observed. The fitted curve for the 0.09 M NH_3_ treatment was concave (*P* = 1.46), whereas the 0.18 and 0.26 M NH_3_ treatment curves were convex (*P* < 1) (Fig. 2C to E). Overall, the values of *δ* (time [h] to the first decimal reduction of the microbial population) and *p* (shape of the inactivation curve) decreased as the level of un-ionized ammonia increased. The 0.26 M NH_3_ treatment had the lowest *δ* and *p* values of 0.17 and 0.49, respectively (Table 2).

**TABLE 2:** Goodness-of-fit measures and parameter values of the fitted models describing the survival of Salmonella Typhimurium in un-ionized ammonia solutions

Treatment	Model	AIC^*a*^^,^^*b*^	BIC^*a*^^,^^*b*^	*N*_0_ ^*a*^ ^,^ ^*b*^	*δ* ^*a*^ ^,^ ^*b*^	*p* ^*a*^ ^,^ ^*b*^	*α* ^*a*^ ^,^ ^*b*^	*δ*_1_ ^*a*^ ^,^ ^*b*^	*δ*_2_ ^*a*^ ^,^ ^*b*^	*N*_res_ ^*a*^ ^,^ ^*b*^
CHES, pH 9	Weibull with tailing effects	−53.32	−50.49	7.93 (7.78, 8.06)	5.73 (4.82, 6.60)	1.12 (0.97, 1.27)	NA	NA	NA	3.62 (3.31, 3.78)
0.04 M NH_3_	Double Weibull	−57.86	−54.32	7.66 (7.53, 7.77)	NA	1.85 (1.70, 2.01)	6.16 (5.79, 6.49)	4.81 (4.42, 5.21)	21.41 (18.10, 26.23)	NA
0.09 M NH_3_	Weibull	−33.97	−33.38	7.77 (7.67, 7.90)	2.97 (2.74, 3.23)	1.46 (1.39, 1.55)	NA	NA	NA	NA
0.18 M NH_3_	Weibull	−52.95	−52.36	7.73 (7.67, 7.77)	0.42 (0.38, 0.45)	0.61 (0.59, 0.62)	NA	NA	NA	NA
0.26 M NH_3_	Weibull	−39.21	−38.62	8.06 (7.93, 8.14)	0.17 (0.13, 0.21)	0.49 (0.46, 0.52)	NA	NA	NA	NA

a Best-fit parameter values and their 95% confidence intervals (in parentheses) are reported.

b AIC, Akaike information criterion; BIC, Bayesian information criterion; *N*_0_, initial inoculum concentration (log CFU/g); *δ*, time (h) to first decimal reduction; *p*, shape of inactivation curve; *α*, fraction of the first subpopulation remaining in the total population; *δ*_1_ and *δ*_2_, time (h) to first decimal reduction of first and second subpopulation, respectively; *N*_res_, starting point of tail (log CFU/g); NA, not applicable.

### Urea, urease, and Corynebacterium urealyticum treatments.

**FIG 3:**
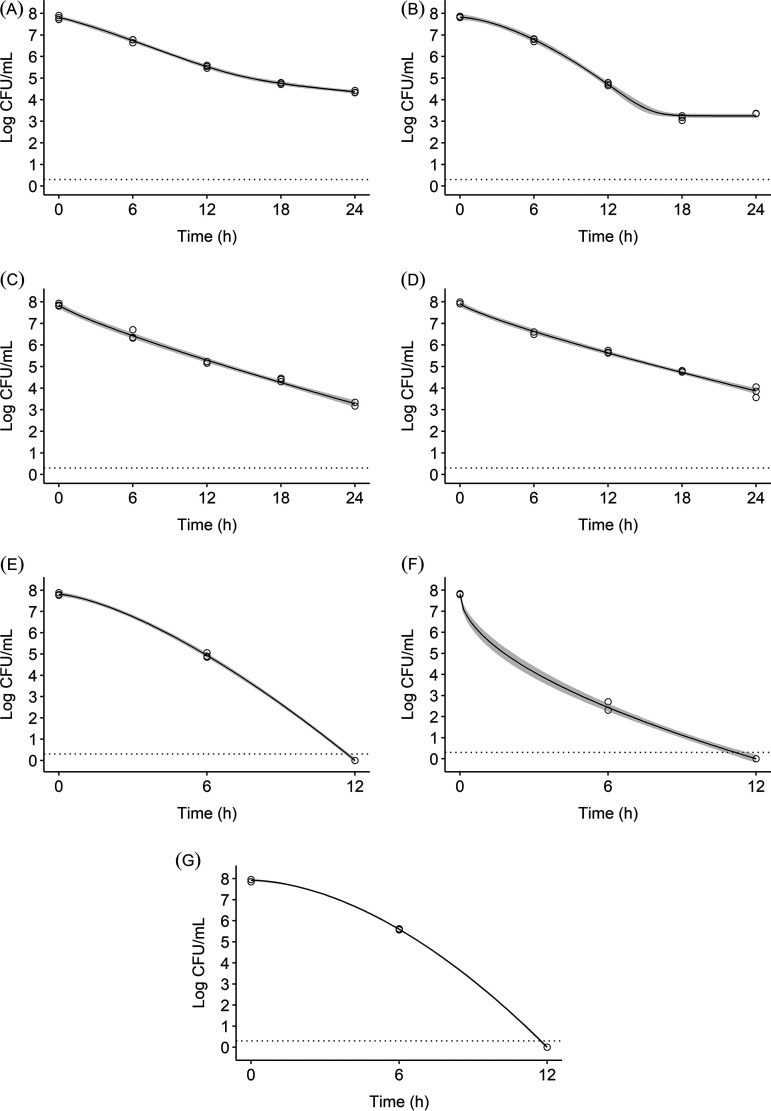
Salmonella Typhimurium survival in urea (1% and 2%, wt/vol), urease, and Corynebacterium urealyticum treatments fitted by Weibull models for urease control (A), *C. urealyticum* control (B), 1% urea (C), 2% urea (D), 1% urea + urease (E), 2% urea + urease (F), and *C. urealyticum *+ 1% urea (G) treatments. Data points for each sampling time (*n* = 3), Weibull model curves, and their 95% confidence intervals (shaded area) are shown. The dotted line indicates the LOD (0.30 log CFU/mL).

The survival of ST in CHES buffer solutions (pH 9) with urea (1% and 2%), urease, and *C. urealyticum* (CU) is shown in Fig. 3. The mean ST populations (log CFU/mL), CU populations (log CFU/mL), pH, and TAN (ppm) at each sampling time are reported in Tables S3, S4, S5, and S6, respectively. The un-ionized ammonia concentrations (M NH_3_) at each sampling time are reported in Table 3. After 24 h, ST populations in the 1% and 2% urea and urease control treatments were reduced to 3.23, 3.83, and 4.37 log CFU/mL, respectively (Fig. 3A, C, and D). Both treatments with urea (1% and 2%) and urease fell below the LOD at 12 h, representing 7.81- and 7.82-log reductions, respectively (Fig. 3E and F). At 12 h, the mean un-ionized ammonia concentrations in the 1% and 2% urea with urease treatments were 0.153 and 0.316 M NH_3_, respectively. After 24 h, the 1% and 2% urea with urease treatments produced un-ionized ammonia concentrations of 0.165 and 0.375 M NH_3_, respectively (Table 3). In the CU with 1% urea treatment, the rate of ammonia production remained constant at approximately 0.04 M NH_3_ every 6 h until 18 h, when this rate decreased (Table 3). ST populations were reduced to 3.36 log CFU/mL in the CU control treatment after 24 h. In the CU with 1% urea treatment, ST populations fell below the LOD after 12 h (7.92-log reduction) (Fig. 3G). The mean un-ionized ammonia concentrations in this treatment were 0.083 and 0.130 M NH_3_ at 12 and 24 h, respectively (Table 3). Ammonia production in the urea with urease and *C. urealyticum* with urea treatments increased the pH up to 9.25 (Table S5). NH_3_ did not have a major effect on the CU inoculum, which decreased by only 1.66 log CFU/mL after 24 h (Table S4). The un-ionized ammonia concentrations in the urea, urease, and CU control treatments were less than 0.001 M NH_3_ at each sampling time (Table 3).

**TABLE 3:** Un-ionized ammonia concentration (M NH_3_) in urea (1% and 2%, wt/vol), urease, and Corynebacterium urealyticum treatments

	Time (h)^*a*^				
Treatment	0	6	12	18	24
Urease control	<0.001 C	<0.001 D	<0.001 D	<0.001 D	<0.001 D
*C. urealyticum* control	<0.001 C	<0.001 D	<0.001 D	<0.001 D	<0.001 D
Urea (1%)	<0.001 C	<0.001 D	<0.001 D	<0.001 D	<0.001 D
Urea (2%)	<0.001 C	<0.001 D	<0.001 D	<0.001 D	<0.001 D
Urea (1%) + urease	0.017 ± 0.002 B	0.139 ± 0.001 B	0.153 ± 0.002 B	0.159 ± 0.002 B	0.165 ± 0.001 B
Urea (2%) + urease	0.020 ± 0.000 A	0.180 ± 0.014 A	0.316 ± 0.002 A	0.371 ± 0.004 A	0.375 ± 0.005 A
*C. urealyticum *+ 1% urea	<0.001 C	0.039 ± 0.001 C	0.083 ± 0.001 C	0.121 ± 0.001 C	0.130 ± 0.003 C

a Data are reported as mean ± standard deviation (*n* = 3). For <0.001, actual values are <1 × 10^−3^ M NH_3_. Un-ionized ammonia concentration was calculated using equations 1 and 2 at 30°C, and pH was measured at each sampling time. Means with different letters in the same column are significantly different (*P* < 0.05).

Table 4 shows parameter estimates, 95% confidence intervals, and goodness-of-fit measures (AIC and BIC) for the fitted Weibull curves of ST survival in urea (1% and 2%), urease, and CU solutions. ST survival in the urease control solution was best fitted to the double Weibull model, where the survival curve was slightly concave (*P* = 1.16) with a minor inflection point between 12 and 18 h (Fig. 3A). The CU control was fitted well to both the trilinear (AIC: −66.54; BIC: −63.71) and Weibull model with tailing effects (AIC: −64.93; BIC: −62.09). With only slight differences between AIC and BIC, the Weibull model with tailing effects was selected to allow for comparisons with the other treatments. The resulting model for the CU control treatment was concave (*P* = 1.59) with the tailing effect (*N*_res_) starting at 3.25 log CFU/mL (Fig. 3B). The Weibull model, without shoulder or tailing effects, was best fitted to ST inactivation data for the urea (1% and 2%) controls and 2% urea with urease treatment. For the 1% urea with urease and CU with 1% urea treatments, identical AIC and BIC values indicated three best-fitting models: the Weibull, Geeraerd with shoulder effects, and bilinear with shoulder effects models. The Weibull model was selected as the preferred model. The 1% and 2% urea controls were both fitted to convex (*P* < 1) survival curves with *δ* values of 3.89 and 4.38, respectively (Fig. 3C and D). The urease and CU treatments with 1% urea were both fitted by concave (*P* > 1) survival curves with *δ* values of 2.88 and 3.73, respectively (Fig. 3E and G). The 2% urea with urease treatment showed the most rapid decline of Salmonella among this group of treatments, with *δ* and *p* values of 0.26 and 0.54, respectively (Fig. 3F and Table 4).

**TABLE 4:** Goodness-of-fit measures and parameter values of the fitted models describing the survival of Salmonella Typhimurium in urea (1% and 2%, wt/vol), urease, and Corynebacterium urealyticum treatments

Treatment	Model	AIC^*a*^^,^^*b*^	BIC^*a*^^,^^*b*^	*N*_0_ ^*a*^ ^,^ ^*b*^	*δ* ^*a*^ ^,^ ^*b*^	*p* ^*a*^ ^,^ ^*b*^	*α* ^*a*^ ^,^ ^*b*^	*δ*_1_ ^*a*^ ^,^ ^*b*^	*δ*_2_ ^*a*^ ^,^ ^*b*^	*N*_res_ ^*a*^ ^,^ ^*b*^
Urease control	Double Weibull	−75.72	−72.18	7.81 (7.74, 7.87)	NA	1.16 (1.02, 1.30)	2.31 (1.94, 2.84)	5.60 (5.16, 6.03)	21.59 (16.73, 39.21)	NA
*C. urealyticum* control	Weibull with tailing effects	−64.93	−62.09	7.83 (7.71, 7.90)	5.86 (5.29, 6.37)	1.59 (1.41, 1.77)	NA	NA	NA	3.25 (3.17, 3.32)
Urea (1%)	Weibull	−56.46	−54.34	7.86 (7.75, 8.02)	3.89 (3.25, 4.55)	0.84 (0.77, 0.91)	NA	NA	NA	NA
Urea (2%)	Weibull	−60.63	−58.50	7.94 (7.79, 8.03)	4.38 (3.78, 5.11)	0.82 (0.76, 0.90)	NA	NA	NA	NA
Urea (1%) + urease	Weibull	−45.14	−44.54	7.81 (7.76, 7.89)	2.88 (2.77, 3.01)	1.44 (1.40, 1.48)	NA	NA	NA	NA
Urea (2%) + urease	Weibull	−34.02	−33.42	7.82 (7.73, 7.95)	0.26 (0.20, 0.35)	0.54 (0.50, 0.58)	NA	NA	NA	NA
*C. urealyticum *+ 1% urea	Weibull	−56.57	−55.97	7.92 (7.87, 7.95)	3.73 (3.66, 3.81)	1.77 (1.74, 1.80)	NA	NA	NA	NA

a Best-fit parameter values and their 95% confidence intervals (in parentheses) are reported.

b AIC, Akaike information criterion; BIC, Bayesian information criterion; *N*_0_, initial inoculum concentration (log CFU/g); *δ*, time (h) to first decimal reduction; *p*, shape of inactivation curve; *α*, fraction of the first subpopulation remaining in total population; *δ*_1_ and *δ*_2_, time (h) to first decimal reduction of first and second subpopulation, respectively; *N*_res_, starting point of tail (log CFU/g); NA, not applicable.

## DISCUSSION

Several studies have demonstrated the antimicrobial effects of NH_3_ on Salmonella in poultry litter and solutions (19, 28, 31, 33–35). However, previous work in poultry litter has often been performed using NH_3_ gas. As a result, key aspects of the interaction between NH_3_ and Salmonella are not fully understood. This research sought to examine these aspects by characterizing the pH dependency, minimum antimicrobial concentration, and efficacy of NH_3_ production in buffer solutions inoculated with *S*. Typhimurium (ST).

Payne et al. (37) observed the growth of Salmonella in poultry litter at pH 7 and 9 under favorable conditions (i.e., *a_w_* and temperature). Several poultry litter surveys have observed the average pH of litter to be approximately 8 (38–40). In this current study, buffer solutions at pH 5, 7, and 8 with and without ammonium sulfate added showed no detrimental effects on the survival of ST in the solutions. However, at pH 9 with and without ammonium sulfate added (0.4 M TAN), ST populations were significantly reduced. Koziel et al. (19) reported that NH_3_ treatments at low pH levels (4.4 to 4.6) failed to show any antimicrobial effect on *S*. Typhimurium in solution. In their study, Salmonella reductions were not observed in the control solution of PBS at pH 9 (19). Given that the CHES buffer did not exhibit any antimicrobial effects on the inoculum at any lower pH level (5 to 8), with or without the addition of ammonium sulfate, the reduction seen in this current study may be a strain-dependent pH sensitivity. This type of strain-dependent sensitivity to alkaline conditions has been previously observed in a study that tested 99 strains of Salmonella enterica in alkaline solutions (pH 11), where authors reported that reductions ranged from 0 to 5 log CFU/mL after 2 h (41). Despite this pH effect, the addition of ammonium sulfate to the variable pH solutions demonstrated that the antimicrobial efficacy of NH_3_ was dependent on alkaline pH levels (≥9).

Field surveys of poultry litter offer conflicting reports on the relationship between Salmonella and ammonia in litter, with studies observing negative (42), positive (39), or no correlations (40, 43) between ammonia levels and Salmonella prevalence or concentration in litter. However, several laboratory studies in poultry litter and buffer solutions have demonstrated the antimicrobial effects of ammonia (19, 27–32, 35). Himathongkham et al. (35) reported an 8 log CFU/mL reduction of *S.* Typhimurium within 12 h in peptone water (pH 9.18) with 0.21% NH_3_. Koziel et al. (19) tested NH_3_ concentrations of 0.05, 0.1, 0.3, and 0.5 M in PBS adjusted to pH 9. In their study, 0.05 M NH_3_ reduced Salmonella populations by 3.5 log CFU/mL after 24 h. In the present study, the minimum antimicrobial concentration of NH_3_ tested was 0.04 M, which led to a 7.4 log CFU/mL reduction of ST after 24 h. Salmonella populations in the present study were reduced by 7.77 log CFU/mL after 18 h with 0.09 M NH_3_ in solution. Similarly, Koziel et al. (19) observed that 0.1 M NH_3_ in solution reduced Salmonella populations by 8.11 log CFU/mL after 24 h. The higher log reductions observed in this study than those observed by Koziel et al. (19) may have resulted from the pH sensitivity demonstrated by the pH dependency experiments. The alkaline pH of the buffer solutions likely served as an added stressor against Salmonella. Previous studies in poultry litter and manure have used various levels of NH_3_ gas to demonstrate its antimicrobial effects on Salmonella (28, 31, 33). However, these concentrations of NH_3_ gas may not be feasibly generated as a result of natural ammonia production in commercial poultry litter. Ammonia production and volatilization in poultry litter increases significantly at pH levels higher than 8 (15, 25, 44). Turnbull et al. (28) reported that the salmonellacidal efficacy of NH_3_ gas increased as the *a_w_* of the litter approached 1.00. Given the pH dependency and the potential effects of *a_w_*, the minimum antimicrobial concentration of NH_3_ in poultry litter may differ considerably from the level reported in this study of buffer solutions.

Schefferle (45) demonstrated that strains of *Corynebacterium*, *Nocardia*, *Streptomyces*, Pseudomonas, *Micrococcus*, *Alcaligenes*, *Achromobacter*, and *Cytophaga* recovered from poultry litter had the ability to break down uric acid and urea into ammonia. Urease-producing bacteria may be present in litter at levels of 6 to 8 log CFU/g (46). *Corynebacterium* represent a major genera of urease-producing bacteria commonly found in poultry litter, and the species Corynebacterium urealyticum has been previously identified in litter samples (45, 47–49). At present, urease-producing bacteria have not been studied in relation to the prevalence or concentration of Salmonella in poultry litter. A recent study by Bucher et al. (50) reported that *S*. Heidelberg concentrations in poultry litter microcosms exhibited a negative correlation with *Nocardiopsis* and a positive correlation with *Bacillaceae* in the litter. They also isolated a consortium of five Bacillus subtilis strains from the poultry litter that reduced the growth of *S*. Heidelberg in a poultry litter extract solution by an average 0.2 log CFU/mL (50). In the present study, CU had no antagonistic effects on the ST inoculum. The constant rate of ammonia production in the CU with 1% urea treatment suggests that urease enzyme activity in the CU population reached its maximum within the first 6 h of the experiment and that the enzymes were fully saturated by urea from 0 to 18 h. At each sampling time, the CU treatment produced less NH_3_ than the urease treatment, suggesting that the CU inoculum produced a urease concentration of <50 U/mL, the concentration of urease added directly into the other treatments. The results of this study demonstrated that urease and *C*. *urealyticum* can produce NH_3_ in solution under these conditions (pH 9, 30°C) and effectively reproduce the antimicrobial activity observed in the previous experiments. Further investigations are needed to examine the relationship between urease-producing bacteria and Salmonella survival in poultry litter.

Previous studies modeling the survival of Salmonella in poultry litter have examined the effects of heat treatments, storage time, and storage conditions using stacked or composted litter (51–53). In these various studies, Chen et al. (51–53) used the exponential, Geeraerd (with shoulder and/or tailing effects) (54), biphasic (55), and double Weibull models (56) to describe the inactivation kinetics of Salmonella in litter. Payne et al. (37) used the exponential and Churchill models (57) to describe the growth and inactivation of Salmonella in poultry litter under various pH and *a_w_* conditions. Whereas the current study compared seven models to determine the best fit, these previous studies only used one or two models to fit their inactivation data. The results of this study demonstrated that the inactivation kinetics of *S*. Typhimurium in NH_3_ solutions were well described by the Weibull, double Weibull, and Weibull with tailing effects models. The CHES (pH 9) and CU control treatments were both fitted to the Weibull model with tailing effects. Tailing in survival curves typically implies either the existence of a more resistant subpopulation of cells or that cells are adapting to the stress during exposure (58). The urease control and 0.04 M NH_3_ treatments were fitted by the double Weibull model. The double Weibull model assumes that the microbial population is composed of two groups with different resistance levels toward a stress treatment (56). Except for the 0.04 M NH_3_ treatment, all NH_3_ and NH_3_-producing treatments were best fit to the Weibull model, where *δ* and *p* values generally decreased as NH_3_ concentrations increased. At NH_3_ levels of ≥0.9 M, Salmonella populations fell below the LOD within 12 h. The use of three time points (0, 6, and 12 h) to fit several treatment models represents a significant limitation of this current study. Given that Salmonella populations may have fallen below the LOD between 6 and 12 h in these treatments, additional observations within these times would likely change the model parameters. Koziel et al. (19) encountered a similar limitation in their study of Salmonella in NH_3_ solutions, where treatments of ≥0.1 M NH_3_ likely fell below their LOD between sampling times of 8 and 24 h. Despite this issue, the modeling results of this study still provide useful information about the behavior of Salmonella under the stress of NH_3_ and the relevant antimicrobial levels of NH_3_ in solutions. Overall, this modeling effort may serve as a primer for further studies on the interactions between Salmonella and NH_3_ in poultry litter and solutions.

In conclusion, this study provides a fundamental understanding of the production of NH_3_, its pH dependency, and the response of Salmonella to various concentrations of NH_3_ in solution. This study observed that the antimicrobial activity of ammonia was dependent on alkaline conditions (pH ≥ 9), where NH_3_ concentrations of ≥0.04 M resulted in significant Salmonella reductions. Urease and *C*. *urealyticum* were shown to be capable of rapidly breaking down urea into ammonia under alkaline conditions, replicating this antimicrobial effect. The results of this study suggest that high NH_3_ levels in poultry litter may reduce the risk of Salmonella contamination in this biological soil amendment. However, future laboratory and field studies are needed to determine the feasibility of NH_3_ demonstrating this salmonellacidal effect in commercial poultry litter environments, given the need for alkaline conditions and sufficient ammonia production.

## MATERIALS AND METHODS

### Bacterial strains and inoculum preparation.

A Salmonella enterica serovar Typhimurium (ST) isolate (AG100R) previously recovered from poultry litter (38) was used to inoculate the solutions in this study. Antimicrobial resistance to 200 μg/mL rifampicin (RIF; Sigma-Aldrich, St. Louis, MO) was induced in the ST isolate using stepwise exposures. The urease-producing bacteria Corynebacterium urealyticum ATCC 43044 (CU) was also used in this study. This urease-producing bacterium has been found naturally in poultry litter (47). To facilitate recovery and enumeration of CU, resistance to 120 μg/mL ampicillin (AMP; Fisher Scientific, Fair Lawn, NJ) was induced using stepwise exposures. To prepare the ST inoculum, a frozen culture was transferred to 10 mL of tryptic soy broth (TSB; BD, Difco, Sparks, MD) with 80 μg/mL RIF and incubated at 37°C for 18 to 24 h in a shaking incubator at 100 rpm. This overnight culture was transferred once more into 10 mL of TSB with 80 μg/mL RIF and incubated under the same conditions. The final culture was prepared by transferring the overnight culture to 25 mL of TSB with 80 μg/mL RIF in a 50-mL conical centrifuge tube (Fisher Scientific) and incubating under the same conditions. Similarly, the CU inoculum was prepared using Mueller-Hinton broth (MHB; BD, Difco) with 10% rabbit serum (Gibco, Dublin, Ireland) and 80 μg/mL AMP as the culture broth. Cells were collected by centrifugation (1,789 × *g*, 10 min) and washed twice in 0.5 M *N*-cyclohexyl-2-aminoethanesulfonic acid (CHES; Acros Organics, Fair Lawn, NJ) buffer or phosphate-buffered saline (PBS; MP Biomedicals, Irvine, CA). The CHES buffer and PBS washing solutions were adjusted to pH 9.0, 8.0, 7.0, or 5.0 using 10 N NaOH or 6 N HCl, depending on the desired pH of each treatment. Enumeration of the ST inoculum was performed by spreading 0.1 mL of 0.1% peptone water (PW; BD, Difco) serial dilutions onto tryptic soy agar (TSA; BD, Difco) with 80 μg/mL RIF, in duplicate, and incubating at 37°C for 18 to 24 h before counting. The CU inoculum was enumerated similarly, using Mueller-Hinton agar (MHA; BD, Difco) with 10% rabbit serum and 80 μg/mL AMP as the plating medium and incubated at 37°C for 48 h. Each bacterial inoculum yielded a final average concentration of approximately 9 log CFU/mL.

### Ammonia, urea, and control treatments.

All the treatments performed in this study are shown in Table 5. No ammonia or urea was added to control treatments. Solutions for the pH and ammonia trials were prepared using ammonium sulfate [(NH_4_)_2_SO_4_; Fisher Scientific] dried in an oven for 1 h at 100°C. In the pH trial, 26.19 g/L (NH_4_)_2_SO_4_ was added to autoclaved CHES or PBS buffers to achieve 0.4 M nominal concentrations of total ammonia nitrogen (TAN). The calculated corresponding concentrations of un-ionized ammonia (NH_3_) in each pH trial solution are shown in Table 1. For the ammonia trial, solutions were prepared by adding dried (NH_4_)_2_SO_4_ to autoclaved CHES buffer to achieve 0.04, 0.09, 0.18, 0.26, and 0.35 M nominal concentrations of NH_3_. Table 6 shows the amounts of (NH_4_)_2_SO_4_ added to achieve the desired TAN and NH_3_ concentrations in each solution. Urea solutions (1% and 2%, wt/vol) were prepared by adding urea to bottles of autoclaved CHES buffer. Urease solutions were prepared to a concentration of 50 U/mL by adding 0.025 g of urease from *Canavalia ensiformis* (jack bean) (specific activity of 40,150 U/g; Sigma-Aldrich) to 20 mL of CHES. The pH of each solution was adjusted to the desired pH for each treatment, as indicated in Table 5, using 10 N NaOH or 6 N HCl. For all treatments, except those with urease or CU inoculum, 9 mL of each solution was transferred to five 15-mL screw-cap conical centrifuge tubes (Fisher Scientific), followed by the addition of 1 mL of ST inoculum. For treatments with urease, 8 mL of each solution was transferred to five 15-mL screw-cap conical centrifuge tubes, followed by the addition of 1 mL of both the urease solution and ST inoculum. For urease-producing bacteria treatments, 8 mL of each solution was transferred to five 15-mL screw-cap conical centrifuge tubes, followed by the addition of 1 mL of both the CU and ST inoculum. All tubes were incubated in a shaking incubator at 30°C and 120 rpm. All treatments were performed in triplicate.

**TABLE 5:** Summary of treatments, buffer solutions, and pH for each experiment

Exptl variable	Treatments^*a*^	Buffer solution	pH
pH	pH controls	PBS	5.0, 7.0
	pH controls	CHES	8.0, 9.0
	0.4 M TAN	PBS	5.0, 7.0
	0.4 M TAN	CHES	8.0, 9.0^*b*^
Un-ionized ammonia	0.04 M NH_3_	CHES	9.0
	0.09 M NH_3_	CHES	9.0
	0.18 M NH_3_	CHES	9.0^*b*^
	0.26 M NH_3_	CHES	9.0
	0.35 M NH_3_	CHES	9.0
Urea and urease	Urease control	CHES	9.0
	Urea (1%)	CHES	9.0
	Urea (2%)	CHES	9.0
	Urea (1%) + urease	CHES	9.0
	Urea (2%) + urease	CHES	9.0
Urease-producing bacteria	Corynebacterium urealyticum control	CHES	9.0
	*C. urealyticum *+ 1% urea	CHES	9.0

a All treatments were performed in triplicate (*n* = 3) at 30°C.

b These two treatments are identical and represent the same data set.

**TABLE 6:** Calculated total ammonia nitrogen (TAN) and un-ionized ammonia (NH_3_) concentrations in ammonia solutions experiment

Treatments	(NH_4_)_2_SO_4_ (g/L)	TAN (M)	TAN (ppm)	NH_3_ (M)^*a*^	NH_3_ (ppm)^*a*^
0.04 M NH_3_	6.55	0.0991	1,388	0.044	751
0.09 M NH_3_	13.10	0.1982	2,776	0.088	1,501
0.18 M NH_3_	26.19	0.3964	5,552	0.176	3,002
0.26 M NH_3_	39.29	0.5946	8,328	0.264	4,504
0.35 M NH_3_	52.38	0.7928	11,105	0.353	6,005

a Calculated using equations 1 and 2, at 30°C and pH 9.

### Sampling procedure.

One tube from each treatment was sampled at each sampling time (0, 6, 12, 18, and 24 h). Bacterial enumeration was performed by transferring 1 mL from the experimental tube to 9 mL of 0.1% PW, serially diluting, and spread plating 0.1 mL onto TSA with 80 μg/mL RIF for ST and plating onto MHA with 10% rabbit serum and 80 μg/mL AMP for CU. As ST and CU populations approached the limit of detection (LOD) (1 CFU/mL), four 250-μL subsamples from the treatment tubes were plated onto TSA with 80 μg/mL RIF and MHA with 10% rabbit serum and 80 μg/mL AMP, respectively. Plates were incubated at 37°C for 18 to 24 h and 48 h for ST and CU, respectively. All treatment tubes were filter sterilized (product number 430320; Corning, Corning, NY) before measuring pH and TAN. For urease and urease-producing bacteria treatments, pH was measured after filtration, and filtrates were stored at −20°C to stop urease activity until TAN was measured. The pH (model HI72911B, Hanna Instruments, Smithfield, RI) and TAN (model HI4101, Hanna Instruments) of each tube were measured using a pH/oxidation-reduction potential (ORP)/ion-selective electrode (ISE) meter (model HI98191, Hanna Instruments). TAN was measured in parts per million according to the manufacturer’s instructions for the ammonia combination ISE. During the pH trial, PBS buffer treatments (pH 5.0 and 7.0), with and without ammonia added, were measured at 0 and 24 h for pH. For these treatments, TAN was measured in duplicate from samples of the prepared bulk PBS solutions without ammonia added and from tubes sampled at 0 h for treatments with 0.4 M TAN added.

### Un-ionized ammonia calculations.

Un-ionized ammonia (NH_3_) concentrations were calculated using TAN (ppm), pH, and temperature (°C) according to the following equations (equations 1 and 2) derived from the Henderson-Hasselbalch equation (pH = p*K_a_* + log_10_[base]/[acid]) and Emerson et al. (18), where 17/14 is added to convert ammonia nitrogen (NH_3_-N) to un-ionized ammonia (NH_3_).

(1) $${\text{NH}}_{\text{3}}\left(\text{ppm}\right)=\frac{17}{14}\times \frac{\text{TAN}}{\left(1+10^{\left(\text{p}K_{a}-\text{pH}\right)}\right)}$$

(2) $$\text{p}K_{a}=0.09018+(\frac{2,729.92}{273.15+{}^{\circ}\text{C }})$$

### Statistical analysis.

Bacterial plate counts (CFU/mL) were log transformed (log_10_ [CFU/mL + 1]) for statistical analysis. The log-transformed LOD was 0.30 log CFU/mL (log_10_ [1 + 1]). Negative samples were assigned a value of 0 CFU/mL and were represented by 0 log CFU/mL (log_10_ [0 + 1]). A one-way analysis of variance (ANOVA) followed by Tukey’s honestly significant difference test was used to compare mean ST populations for all treatments and un-ionized ammonia concentrations (M NH_3_) for the urea, urease, and CU treatments. Welch’s *t* test was used to compare mean CU populations at each sampling time. Statistical analyses were performed in R version 4.0.4 (59) with a significance level of 0.05.

### Inactivation models, parameter estimation, and goodness-of-fit.

Several primary inactivation models were fitted to the survival data to determine the best-fitting model. The following models were considered: log-linear (60), bilinear (with and without tailing or shoulder effects) (61), trilinear (61), Geeraerd (with and without tailing or shoulder effects) (54), Weibull (62), Weibull with tailing effects (63), and double Weibull (56). ST concentration (log CFU/mL) data from the NH_3_, urea, urease, and urease-producing bacteria experiments were fitted to these models using the nls() function in R (59). Bootstrapped confidence intervals were generated for the fitted model parameters using the nlsBoot() function from the nlsMicrobio R package (61). For each treatment, ST concentrations were modeled for sampling times between inoculation (0 h) and the first sample time approaching the LOD. Survival data from the 0.35 M NH_3_ treatment were not modeled because the data could not be fitted reliably. The goodness-of-fit among fitted models was compared according to the Akaike information criterion (AIC; equation 3) (64) and the Bayesian information criterion (BIC; equation 4) (65).

(3) $$\text{AIC}=p\cdot \text{ln}\left(\frac{\mathrm{RSS}}{p}\right)+2k$$

(4) $$\text{BIC}=p\cdot \text{ln}\left(\frac{\mathrm{RSS}}{p}\right)+k\cdot \text{ln}(p)$$

In equations 3 and 4, *RSS* is the residual sum of squares, *p* is the number of data points used to fit the model, and *k* is the number of parameters in the model. Lower AIC and BIC scores indicate a better fitting model.

Inactivation kinetics in this study were described by the Weibull, Weibull with tailing effects, and double Weibull models. The Weibull model (equation 5) is an empirical inactivation model used to fit microbial survival curves that exhibit non-log-linear behavior (66).

(5) $$\log_{10}(N_{t})=\log_{10}(N_{0})-{\left(\frac{t}{\delta }\right)}^{p}$$

In this model (equation 5), *N_t_* is the microbial population (CFU/mL) at time *t*, *N*_0_ is the initial microbial population (CFU/mL), *t* is the time (h), *p* is the shape of the inactivation curve (dimensionless), and *δ* is the time (h) to the first decimal reduction of the microbial population.

Albert and Mafart (63) proposed a modified Weibull model that incorporated parameters for modeling shoulder (delayed response) and/or tailing (stabilized decline) phenomena observed in microbial survival curves. This model is presented here with only tailing incorporated (equation 6).

(6) $$\log_{10}(N_{t})={\text{log}}_{10}\left[(N_{0}-N_{\text{res}})10^{-{\left(\frac{t}{\delta }\right)}^{p}}+N_{\text{res}}\right]$$

In this model (equation 6), *N_t_*, *N*_0_, *t*, *p*, and *δ* have the same meaning as previously described, while *N*_res_ is the microbial population (CFU/mL) at the starting point of the tail, which is supposed to be fully resistant against inactivation.

Coroller et al. (56) proposed a new primary inactivation model based on two mixed Weibull distributions, which could be used to describe survival curves where the microbial population is assumed to be composed of two subpopulations with different levels of resistance to stress (equation 7).

(7) $$\log_{10}(N_{t})=\frac{N_{0}}{1+10^{\alpha }}\left[10^{-{\left(\frac{t}{\delta_{1}}\right)}^{p}+\alpha }+10^{-{\left(\frac{t}{\delta_{2}}\right)}^{p}}\right]$$

In this double Weibull model (equation 7), *N_t_*, *N*_0_, *t*, and *p* have the same meaning as previously described, while *α* is the fraction of the first subpopulation remaining in the total population, and *δ*_1_ and *δ*_2_ are the times to the first decimal reduction of subpopulations 1 and 2, respectively (67).

## Supplementary Material

Reviewer comments
