# Supplementary material for: Antimicrobial Efficacy of Un-Ionized Ammonia (NH3) against Salmonella Typhimurium in Buffered Solutions with Variable pH, NH3 Concentrations, and Urease-Producing Bacteria
Source: Microbiol Spectr. 2022 Jan 19;10(1):e01850-21. doi: 10.1128/spectrum.01850-21 (PMC8768630; doi:10.1128/spectrum.01850-21)
Supplement: SUPPLEMENTAL FILE 1 — Supplemental material. Download SPECTRUM01850-21_Supp_1_seq7.pdf, PDF file, 0.1 MB [file spectrum01850-21_supp_1_seq7.pdf]

SUPPLEMENTAL MATERIAL

Table S1. *Salmonella* Typhimurium concentration (log CFU/mL) in buffer solutions (PBS, CHES) at various pH levels (5, 7, 8, 9), with and without ammonium sulfate added (0.4 M TAN)[a]

Treatment	Time (h)				
	0	6	12	18	24
PBS, pH 5	8.12 ± 0.02 bcd	8.08 ± 0.02 ab	8.07 ± 0.04 a	7.98 ± 0.05 ab	8.04 ± 0.02 a
PBS, pH 7	8.13 ± 0.06 bc	8.05 ± 0.05 ab	8.02 ± 0.03 a	8.38 ± 0.53 a	7.95 ± 0.04 a
CHES, pH 8	8.01 ± 0.01 de	7.95 ± 0.05 bc	7.83 ± 0.05 a	7.74 ± 0.07 b	7.69 ± 0.05 b
CHES, pH 9	7.95 ± 0.04 e	6.79 ± 0.13 d	5.75 ± 0.07 b	4.35 ± 0.20 c	3.70 ± 0.17 d
0.4 M TAN, pH 5	8.25 ± 0.05 a	8.11 ± 0.08 ab	7.96 ± 0.07 a	7.95 ± 0.05 ab	7.99 ± 0.06 a
0.4 M TAN, pH 7	8.20 ± 0.06 ab	8.15 ± 0.06 a	8.10 ± 0.03 a	8.03 ± 0.04 ab	8.01 ± 0.09 a
0.4 M TAN, pH 8	8.05 ± 0.05 cde	7.84 ± 0.06 c	8.46 ± 1.10 a	7.58 ± 0.08 b	7.19 ± 0.07 c
0.4 M TAN, pH 9	7.73 ± 0.01 f	2.66 ± 0.08 e	0.00 ± 0.00 c	0.00 ± 0.00 d	0.00 ± 0.00 e

[a] Reported as mean ± standard deviation (n = 3). Means with different letters in the same column are significantly different ($P < 0.05$).

Table S2. *Salmonella* Typhimurium concentration (log CFU/mL) in CHES buffer (pH 9) with various levels of un-ionized ammonia (M NH_3)[a]

	Time (h)				
Treatment	0	6	12	18	24
CHES, pH 9	7.95 ± 0.04 ab	6.79 ± 0.13 a	5.75 ± 0.07 a	4.35 ± 0.20 a	3.70 ± 0.17 a
0.04 M NH_3	7.66 ± 0.12 c	6.15 ± 0.04 b	2.25 ± 0.05 b	0.77 ± 0.07 b	0.26 ± 0.24 b
0.09 M NH_3	7.77 ± 0.05 bc	4.99 ± 0.14 c	0.10 ± 0.17 c	0.00 ± 0.00 c	0.00 ± 0.00 b
0.18 M NH_3	7.73 ± 0.01 c	2.66 ± 0.08 d	0.00 ± 0.00 c	0.00 ± 0.00 c	0.00 ± 0.00 b
0.26 M NH_3	8.06 ± 0.02 a	2.32 ± 0.17 e	0.00 ± 0.00 c	0.00 ± 0.00 c	0.00 ± 0.00 b
0.35 M NH_3	7.98 ± 0.09 a	0.36 ± 0.10 f	0.93 ± 0.90 c	0.00 ± 0.00 c	0.00 ± 0.00 b

[a] Reported as mean ± standard deviation (n = 3). Means with different letters in the same column are significantly different ($P < 0.05$).

Table S3. *Salmonella* Typhimurium concentration (log CFU/mL) in CHES buffer (pH 9) with urea (1%, 2% w/v), urease, and *Corynebacterium urealyticum*[a]

Treatment	Time (h)				
	0	6	12	18	24
Urease control	7.81 ± 0.09 a	6.74 ± 0.08 a	5.53 ± 0.07 a	4.76 ± 0.04 a	4.37 ± 0.06 a
C. urealyticum control	7.83 ± 0.03 a	6.77 ± 0.07 a	4.72 ± 0.07 b	3.16 ± 0.11 c	3.36 ± 0.01 b
1% urea	7.86 ± 0.06 a	6.46 ± 0.21 a	5.19 ± 0.04 c	4.39 ± 0.08 b	3.23 ± 0.10 b
2% urea	7.94 ± 0.04 a	6.57 ± 0.06 a	5.68 ± 0.06 d	4.77 ± 0.03 a	3.83 ± 0.25 c
1% urea + urease	7.81 ± 0.06 a	4.94 ± 0.11 c	0.00 ± 0.00 e	0.00 ± 0.00 d	0.00 ± 0.00 d
2% urea + urease	7.82 ± 0.02 a	2.44 ± 0.23 d	0.00 ± 0.00 e	0.00 ± 0.00 d	0.00 ± 0.00 d
C. urealyticum + 1% urea	7.92 ± 0.06 a	5.60 ± 0.03 b	0.00 ± 0.00 e	0.00 ± 0.00 d	0.00 ± 0.00 d

[a] Reported as mean ± standard deviation (n = 3). Means with different letters in the same column are significantly different ($P < 0.05$).

Table S4. *Corynebacterium urealyticum* concentration (log CFU/mL) in CHES buffer (pH 9) with and without urea (1% w/v)[a]

Treatment	Time (h)				
	0	6	12	18	24
C. urealyticum control	7.82 ± 0.05 a	7.88 ± 0.02 a	7.58 ± 0.07 a	7.16 ± 0.04 a	6.63 ± 0.11 a
C. urealyticum + 1% urea	7.83 ± 0.25 a	7.44 ± 0.05 b	6.94 ± 0.08 b	6.71 ± 0.13 b	6.17 ± 0.08 b

[a] Reported as mean ± standard deviation (n = 3). Means with different letters in the same column are significantly different ($P < 0.05$).

Table S5. pH measurements for all treatments at each sampling time[a]

Treatment	Time (h)				
	0	6	12	18	24
PBS, pH 5	4.84 ± 0.11	ND	ND	ND	4.88 ± 0.08
PBS, pH 7	6.93 ± 0.01	ND	ND	ND	6.95 ± 0.01
CHES, pH 8	8.06 ± 0.01	8.02 ± 0.01	8.05 ± 0.01	8.02 ± 0.01	8.02 ± 0.00
CHES, pH 9	9.10 ± 0.01	9.05 ± 0.00	9.07 ± 0.00	9.07 ± 0.00	9.07 ± 0.00
0.4 M TAN, pH 5	4.89 ± 0.04	ND	ND	ND	4.91 ± 0.08
0.4 M TAN, pH 7	6.95 ± 0.02	ND	ND	ND	6.87 ± 0.01
0.4 M TAN, pH 8	8.07 ± 0.01	7.99 ± 0.01	7.98 ± 0.02	7.98 ± 0.02	7.98 ± 0.01
0.04 M NH_3	9.08 ± 0.01	9.00 ± 0.01	9.06 ± 0.01	9.00 ± 0.01	9.00 ± 0.00
0.09 M NH_3	9.08 ± 0.01	9.06 ± 0.02	9.08 ± 0.01	9.05 ± 0.02	9.04 ± 0.01
0.18 M NH_3[b]	9.09 ± 0.01	9.07 ± 0.01	9.08 ± 0.01	9.06 ± 0.02	9.05 ± 0.02
0.26 M NH_3	9.07 ± 0.01	9.01 ± 0.01	8.95 ± 0.01	8.96 ± 0.02	8.94 ± 0.02
0.35 M NH_3	9.08 ± 0.01	9.01 ± 0.02	8.96 ± 0.02	8.95 ± 0.02	8.94 ± 0.02
Urease control	9.10 ± 0.00	9.08 ± 0.02	9.09 ± 0.01	9.06 ± 0.01	9.06 ± 0.01
C. urealyticum control	9.06 ± 0.01	9.04 ± 0.01	9.02 ± 0.01	9.01 ± 0.01	8.99 ± 0.00
1% urea	9.09 ± 0.01	9.04 ± 0.00	9.04 ± 0.00	9.04 ± 0.01	9.04 ± 0.00
2% urea	9.07 ± 0.01	9.02 ± 0.01	9.03 ± 0.01	9.03 ± 0.01	9.03 ± 0.01
1% urea + urease	9.04 ± 0.01	9.13 ± 0.01	9.18 ± 0.01	9.19 ± 0.01	9.05 ± 0.01
2% urea + urease	9.05 ± 0.01	9.16 ± 0.01	9.23 ± 0.01	9.25 ± 0.01	9.23 ± 0.00
C. urealyticum + 1% urea	9.08 ± 0.01	9.11 ± 0.01	9.14 ± 0.01	9.16 ± 0.01	9.15 ± 0.01

[a] Reported as mean ± standard deviation (n = 3). ND, not determined.

[b] Treatment is identical to 0.4 M TAN, pH 9, represents the same dataset.

Table S6. Total ammonia nitrogen concentration (ppm TAN) of all treatments at each sampling time[a]

Treatment	Time (h)				
	0	6	12	18	24
PBS, pH 5[c]	0.11 ± 0.01	ND	ND	ND	ND
PBS, pH 7[c]	0.16 ± 0.04	ND	ND	ND	ND
CHES, pH 8	1.81 ± 0.04	1.97 ± 0.05	1.85 ± 0.04	1.81 ± 0.06	1.66 ± 0.05
CHES, pH 9	2.01 ± 0.02	2.09 ± 0.02	2.11 ± 0.02	2.17 ± 0.09	2.17 ± 0.08
0.4 M TAN, pH 5	5,047 ± 80.83	ND	ND	ND	ND
0.4 M TAN, pH 7	5,147 ± 41.63	ND	ND	ND	ND
0.4 M TAN, pH 8	5,020 ± 69.28	4,940 ± 111.36	4,787 ± 94.52	4,920 ± 69.28	4,933 ± 80.83
0.04 M NH_3	1,185 ± 25.79	1,180 ± 7.21	1,173 ± 14.47	1,145 ± 11.02	1,170 ± 7.21
0.09 M NH_3	2,360 ± 20.00	2,287 ± 30.55	2,300 ± 0.00	2,260 ± 34.64	2,193 ± 11.55
0.18 M NH_3[b]	4,447 ± 50.33	4,387 ± 30.55	4,467 ± 30.55	4,353 ± 11.55	4,380 ± 20.00
0.26 M NH_3	7,127 ± 30.55	7,113 ± 41.63	7,267 ± 30.55	7,353 ± 57.74	7,293 ± 120.55
0.35 M NH_3	9,807 ± 57.74	9,807 ± 11.55	9,853 ± 30.55	9,873 ± 61.10	10,000 ± 111.36
Urease control	1.67 ± 0.08	1.58 ± 0.08	1.55 ± 0.02	1.48 ± 0.04	1.47 ± 0.06
C. urealyticum control	2.09 ± 0.08	2.23 ± 0.15	2.27 ± 0.08	2.18 ± 0.17	2.25 ± 0.06
1% urea	1.55 ± 0.01	1.69 ± 0.03	1.69 ± 0.05	1.77 ± 0.16	1.88 ± 0.13
2% urea	1.53 ± 0.11	1.78 ± 0.04	2.01 ± 0.06	2.17 ± 0.04	2.36 ± 0.14
1% urea + urease	511 ± 54.60	3,740 ± 52.92	3,920 ± 20.00	4,000 ± 80.00	4,180 ± 52.92
2% urea + urease	586 ± 3.46	4,720 ± 417.61	7,673 ± 61.10	8,827 ± 117.19	9,113 ± 133.17
C. urealyticum + 1% urea	21.20 ± 0.72	1,093 ± 14.19	2,227 ± 50.33	3,160 ± 0.00	3,433 ± 92.38

[a] Reported as mean ± standard deviation (n = 3). ND, not determined.

[b] Treatment is identical to 0.4 M TAN, pH 9, represents the same dataset.

[c] Measured from duplicate samples (n = 2) of the prepared PBS solutions, not from experimental tubes.

Table S7. Un-ionized ammonia concentration (M NH_3) of pH and ammonia treatments at each sampling time[a]

Treatment	Time (h)				
	0	6	12	18	24
PBS, pH 5[c]	< 0.0001	ND	ND	ND	ND
PBS, pH 7[c]	< 0.0001	ND	ND	ND	ND
CHES, pH 8	< 0.0001	< 0.0001	< 0.0001	< 0.0001	< 0.0001
CHES, pH 9	< 0.0001	< 0.0001	< 0.0001	< 0.0001	< 0.0001
0.4 M TAN, pH 5	< 0.0001	ND	ND	ND	ND
0.4 M TAN, pH 7	0.003 ± 0.000	ND	ND	ND	ND
0.4 M TAN, pH 8	0.031 ± 0.001	0.025 ± 0.001	0.024 ± 0.001	0.025 ±0.001	0.025 ± 0.001
0.04 M NH_3	0.041 ± 0.001	0.038 ± 0.000	0.040 ± 0.001	0.037 ± 0.000	0.037 ± 0.000
0.09 M NH_3	0.083 ± 0.001	0.079 ± 0.001	0.080 ± 0.001	0.077 ± 0.001	0.073 ± 0.001
0.18 M NH_3[b]	0.158 ± 0.001	0.151 ± 0.001	0.157 ± 0.001	0.148 ± 0.003	0.149 ± 0.003
0.26 M NH_3	0.248 ± 0.003	0.229 ± 0.002	0.217 ± 0.003	0.221 ± 0.003	0.215 ± 0.001
0.35 M NH_3	0.343 ± 0.005	0.317 ± 0.006	0.297 ± 0.006	0.294 ± 0.009	0.292 ± 0.006

[a] Reported as mean ± standard deviation (n = 3). Values calculated using TAN, pH, 30°C, and eq. 1 and 2. ND, not determined. < 0.0001, actual values are < 1×10^{-4} M NH_3.

[b] Treatment is identical to 0.4 M TAN, pH 9, represents the same dataset.

[c] Measured from duplicate samples (n = 2) of the prepared PBS solutions, not from experimental tubes.
