# Supplementary material for: Antimicrobial Efficacy of Un-Ionized Ammonia (NH3) against Salmonella Typhimurium in Buffered Solutions with Variable pH, NH3 Concentrations, and Urease-Producing Bacteria
Source: Microbiol Spectr. 2022 Jan 19;10(1):e01850-21. doi: 10.1128/spectrum.01850-21 (PMC8768630; doi:10.1128/spectrum.01850-21)
Supplement: Reviewer comments [file reviewer-comments.pdf]

AMERICAN SOCIETY FOR MICROBIOLOGY | Microbiology Spectrum

Antimicrobial efficacy of un-ionized ammonia (NH_3) against Salmonella Typhimurium in buffered solutions with variable pH, NH_3 concentrations, and urease-producing bacteria

Alan Gutierrez, Arie Havelaar, and Keith Schneider

Corresponding Author(s): Keith Schneider, University of Florida

Review Timeline:

Submission Date:	October 8, 2021
Editorial Decision:	November 23, 2021
Revision Received:	December 18, 2021
Editorial Decision:	December 20, 2021
Revision Received:	December 21, 2021
Accepted:	December 22, 2021

Editor: Sandeep Tamber

Reviewer(s): The reviewers have opted to remain anonymous.

Transaction Report:

(Note: With the exception of the correction of typographical or spelling errors that could be a source of ambiguity, letters and reports are not edited. The original formatting of letters and referee reports may not be reflected in this compilation.)

DOI: https://doi.org/10.1128/Spectrum.01850-21

November 23, 2021

Prof. Keith Schneider
University of Florida
Food Science and Human Nutrition
359 FSHN Bldg.
Newell Dr.
Gainesville, FL 32611

Re: Spectrum01850-21 (Antimicrobial efficacy of un-ionized ammonia (NH_3) against Salmonella Typhimurium in buffered solutions with variable pH, NH_3 concentrations, and urease-producing bacteria)

Dear Prof. Keith Schneider:

Thank you for submitting your manuscript to Microbiology Spectrum. When submitting the revised version of your paper, please provide (1) point-by-point responses to the issues raised by the reviewers as file type "Response to Reviewers," not in your cover letter, and (2) a PDF file that indicates the changes from the original submission (by highlighting or underlining the changes) as file type "Marked Up Manuscript - For Review Only". Please use this link to submit your revised manuscript - we strongly recommend that you submit your paper within the next 60 days or reach out to me. Detailed instructions on submitting your revised paper are below.

Link Not Available

Thank you for the privilege of reviewing your work. Below you will find instructions from the Microbiology Spectrum editorial office and comments generated during the review.

The ASM Journals program strives for constant improvement in our submission and publication process. Please tell us how we can improve your experience by taking this quick Author Survey.

Sincerely,

Sandeep Tamber

Editor, Microbiology Spectrum

Journals Department
American Society for Microbiology
1752 N St., NW
Washington, DC 20036
E-mail: spectrum@asmusa.org

Reviewer comments:

Reviewer #1 (Comments for the Author):

The authors have performed a nice study investigating the behavior of salmonella in solution looking at the effect of ammonia and pH on rate of an activation. This research may inform future applied research on salmonella inactivation in poultry litter.

The work is generally sound, and my comments are generally minor.

There are some issues with reporting samples below the detection limit with being at the detection limit which might have implications for modeling or other conclusions.

I would also note that much of the discussion does not really discuss the published literature and needs to be significantly rewritten or combined with results if that is allowed by the journal.

Page 2:
Content: "and production efficacy of NH3."
Comment: Unclear what this phrase means.

Page 2:
Content: "Salmonella was completely inactivated (~ 8 log CFU/mL reduction)"
Comment: "Completely inactivated" depends upon starting concentration and detection limit. Better just to report the log reduction as the authors do here and delete this phrase throughout the document.

Page 2:
Content: "while 0.04 M NH3 represented the minimum antimicrobial concentration tested (7.40 log CFU/mL reduction after 24 h).
"
Comment: Awkward phrasing. Maybe divide the sentence into two?

Page 2:
Content: "which completely inactivated Salmonella within 12 h."
Comment: This phrase is unscientific and not helpful. Please eliminate throughout the manuscript

Page 2:
Content: "completely inactivated"
Comment: Same comment

Page 3:
Content: "Poultry litter is recognized as an environmental source of Salmonella dissemination among birds in poultry houses (7-9). Poultry litter is a mixture of poultry excreta, feathers, wasted feed, and bedding materials from poultry houses (10)."
Comment: Better to reverse the order of these two sentences. Explain what poultry litter is before talking about why it's a problem.

Page 4:
Content: "S. Typhimurium in poultry litter was reduced by 10 log CFU/mL"
Comment: First, it seems impossible that a log reduction this high could be calculated. Second, poultry litter is measured in grams not mL. Please check the reference and confirm.

Page 9:
Content: "Bacterial plate counts (CFU/mL) were log-transformed [log10 (CFU/mL + 1)] for statistical analysis. When ST populations fell below the LOD (1 CFU/mL; 0 log CFU/mL), samples were assigned a value of 0 CFU/mL."
Comment: This doesn't make sense and it may result in incorrect models.

Page 11:
Content: "The calculated un-ionized ammonia"
Comment: The word un-ionized is also spelled elsewhere in the manuscript without a hyphen. I think the authors should be consistent, and I prefer the hyphenated form.

Page 14:
Content: "DISCUSSION"
Comment: As noted below, a great deal of the discussion is simply recapitulation of results with no discussion of the published literature in the context of the current work. The discussion really needs to be entirely rewritten, or the authors need to write a combined results in discussion section if allowed by the journal format.

Page 15:
Content: "The minimum antimicrobial concentration of NH3 was determined in this study by inoculating ST into CHES buffer, adjusted to pH 9, with varying levels of ammonium sulfate added to achieve 0.04, 0.09, 0.18, 0.26, and 0.35 M NH3 (Table 3). The minimum antimicrobial concentration of NH3 was 0.04 M, which led to a 7.4 log CFU/mL reduction of ST within 24 h (Fig. 2B). "
Comment: These sentences are methods and results, and should be deleted from the discussion.

Page 16:
Content: "The present study examined the mechanisms of NH3 production and the interactions between urea, urease, CU, and the ST inoculum. The urea (1% and 2%), urease, and CU control treatments (pH 9) resulted in Salmonella reductions of 3.4-4.6 log CFU/mL, whereas CHES (pH 9) control resulted in a 4.25-log reduction after 24 h (Fig. 3A-D and 2A)."
Comment: Unfortunately the only sentence in this paragraph which is really discussion is the first two sentences. From the sentence noted above onward to the end of the paragraph is simply a recapitulation of results. The authors make no attempt to discuss their findings in the context of the rest of the literature. This paragraph needs to be rewritten or deleted.

Page 17:
Content: "Modeling was used in this study to describe the inactivation kinetics and assess the microbial stress response of S. Typhimurium to NH3 in solution."
Comment: The paragraph that starts with this sentence is also simply a recapitulation of the results. There is essentially no discussion, therefore this paragraph should be deleted.

Page 18:
Content: "Except for the 0.04 M NH3 treatment, all NH3 and NH3-producing treatments were best fit to the Weibull model."
Comment: This paragraph sites no references, therefore it is not discussion, it is once again a recapitulation of the results and should be deleted.

Page 18:
Content: "Among the NH3 concentration treatments, the δ and p values decreased as the concentration of NH3 was increased."
Comment: This paragraph is almost entirely a recapitulation of results with very little discussion. References are cited in the final sentence, but very little attempt made to discuss them in the context of the current work.

Page 27:
Content: "Experiment "
Comment: This does not seem like the right header for this column in the table. Maybe experimental variable?

Page 33:
Content: "Figure 1. Survival of Salmonella Typhimurium in buffer solutions (PBS, CHES) at various pH levels (5, 7,"
Comment: The results shown in the figure for TAN pH 9 are misleading. If no colonies were recovered at the limit of detection, they should not be plotted on the graph.

Page 34:
Content: "Figure 2."
Comment: In this figure and those following, the authors need to be very careful about reporting results at the limit of detection. If they did not detect any colonies it is misleading to report zero log CFU. This can have profound effects on the model fitting, especially since data were only collected at three time points. If the last time point no colonies were detected, then two points determine a straight line. If the authors had chosen a different final sampling time and also found no colonies, this could change the model significantly.

Reviewer #2 (Comments for the Author):

The authors of this study suggest that ammonia in poultry litter may affect the survival of Salmonella. This is an important topic and over all the manuscript is informative. The breakdown of urea to ammonia or other products can occur in various ways in the environment. Some confusion in the manuscript relates to this complex system. Overall, the results and discussion are clear, but the introduction is less clear. Perhaps the experiment can be set up and better described in the introduction.

The application and direction of study is unclear. Are the authors describing ammonia as if it would be a result or aspect of amended soils? Or are the authors describing ammonia gas produced during poultry production, in which case the age of the birds and the history of the house clean out are critical points of information. If this is a more basic study, as the title may suggest, then this should be clarified. The introduction seems to contain relevant information from all areas.
The story of ammonia and urea in soils is complicated, and the gaseous ammonia story can also be complicated, so the authors should be clear in terms of the narrative being written with the introduction.

Line 41-42 of the Importance could be reworded, as a sentence like "Previous studies..." should have references to support it, but references are not typically included in the Importance section.

Lines 63-78 include an excellent discussion about the breakdown of ammonia. It should be made clear that ammonia transmission and breakdown in the soil is different from gaseous ammonia. It is not clear if this is true or not based on the writings here. Likewise in lines 84-85 the authors are discussing gaseous ammonia being applied to poultry houses.
There should be a discussion of breakdown of ammonia and ammonium salts in the soil.

The authors should be clear about the spelling and writing of the word "unionized".

Staff Comments:

Preparing Revision Guidelines
To submit your modified manuscript, log onto the eJP submission site at https://spectrum.msubmit.net/cgi-bin/main.plex. Go to Author Tasks and click the appropriate manuscript title to begin the revision process. The information that you entered when you first submitted the paper will be displayed. Please update the information as necessary. Here are a few examples of required updates that authors must address:

• Point-by-point responses to the issues raised by the reviewers in a file named "Response to Reviewers," NOT IN YOUR COVER LETTER.
• Upload a compare copy of the manuscript (without figures) as a "Marked-Up Manuscript" file.
• Each figure must be uploaded as a separate file, and any multipanel figures must be assembled into one file.
• Manuscript: A .DOC version of the revised manuscript
• Figures: Editable, high-resolution, individual figure files are required at revision, TIFF or EPS files are preferred

For complete guidelines on revision requirements, please see the journal Submission and Review Process requirements at https://journals.asm.org/journal/Spectrum/submission-review-process. **Submissions of a paper that does not conform to Microbiology Spectrum guidelines will delay acceptance of your manuscript.** "

Please return the manuscript within 60 days; if you cannot complete the modification within this time period, please contact me. If you do not wish to modify the manuscript and prefer to submit it to another journal, please notify me of your decision immediately so that the manuscript may be formally withdrawn from consideration by Microbiology Spectrum.

If your manuscript is accepted for publication, you will be contacted separately about payment when the proofs are issued; please follow the instructions in that e-mail. Arrangements for payment must be made before your article is published. For a complete list of **Publication Fees**, including supplemental material costs, please visit ourwebsite.

Corresponding authors may join or renew ASM membership to obtain discounts on publication fees. Need to upgrade your membership level? Please contact Customer Service at Service@asmusa.org.

Thank you for submitting your paper to Microbiology Spectrum.

Reviewer comments:

Reviewer #1 (Comments for the Author):

The authors have performed a nice study investigating the behavior of salmonella in solution looking at the effect of ammonia and pH on rate of an activation. This research may inform future applied research on salmonella inactivation in poultry litter.

The work is generally sound, and my comments are generally minor.

There are some issues with reporting samples below the detection limit with being at the detection limit which might have implications for modeling or other conclusions.

I would also note that much of the discussion does not really discuss the published literature and needs to be significantly rewritten or combined with results if that is allowed by the journal.

The authors appreciate the reviewers' detailed comments and the opportunity to improve the manuscript for further consideration. The limit of detection has been clarified in the methods section and all figures have been corrected to accurately show the limit of detection. In addition, the discussion has been significantly re-worked to improve its clarity and quality for the reader.

Page 2:
Content: "and production efficacy of NH3."
Comment: Unclear what this phrase means.

This phrase has been revised to improve clarity.

Page 2:
Content: "Salmonella was completely inactivated (~ 8 log CFU/mL reduction)"
Comment: "Completely inactivated" depends upon starting concentration and detection limit. Better just to report the log reduction as the authors do here and delete this phrase throughout the document.

This term has been removed and replaced with the log reduction value.

Page 2:
Content: "while 0.04 M NH3 represented the minimum antimicrobial concentration tested (7.40 log CFU/mL reduction after 24 h). "
Comment: Awkward phrasing. Maybe divide the sentence into two?

This statement has been split into another sentence to improve clarity.

Page 2:
Content: "which completely inactivated Salmonella within 12 h."
Comment: This phrase is unscientific and not helpful. Please eliminate throughout the manuscript

This term has been removed and revised to remain within the abstract word limit.

Page 2:
Content: "completely inactivated"
Comment: Same comment

This term has been removed and revised to remain within the abstract word limit.

Page 3:
Content: "Poultry litter is recognized as an environmental source of Salmonella dissemination among birds in poultry houses (7-9). Poultry litter is a mixture of poultry excreta, feathers, wasted feed, and bedding materials from poultry houses (10)."
Comment: Better to reverse the order of these two sentences. Explain what poultry litter is before talking about why it's a problem.

These sentences have been reversed at the reviewer's suggestion to improve the readability of the manuscript.

Page 4:
Content: "S. Typhimurium in poultry litter was reduced by 10 log CFU/mL"
Comment: First, it seems impossible that a log reduction this high could be calculated. Second, poultry litter is measured in grams not mL. Please check the reference and confirm.

Turnbull and Snoeyenbos (1973) specify that their inoculum was 10^{10} cells/mL. They used standard plating methods to recover *Salmonella* from the litter and all figures use the units of log cells/mL. Certainly, in a modern context, this high inoculum level may seem unusual. Therefore, the authors have chosen to adjust this reference in a way that remains factual, yet less peculiar to modern readers.

Page 9:
Content: "Bacterial plate counts (CFU/mL) were log-transformed [log10 (CFU/mL + 1)] for statistical analysis. When ST populations fell below the LOD (1 CFU/mL; 0 log CFU/mL), samples were assigned a value of 0 CFU/mL."
Comment: This doesn't make sense and it may result in incorrect models.

This statement has been revised to correct the explanation of the log-transformed LOD and negative samples. Figures have also been revised to the correct LOD of 0.30 log CFU/mL [$\log_{10}$ (1 + 1)] according to the method described. This method was used to avoid the use of a negative log scale for censored values of negative samples.

Page 11:
Content: "The calculated un-ionized ammonia"
Comment: The word un-ionized is also spelled elsewhere in the manuscript without a hyphen. I think the authors should be consistent, and I prefer the hyphenated form.

This has been corrected to a consistent spelling of "un-ionized".

Page 14:
Content: "DISCUSSION"
Comment: As noted below, a great deal of the discussion is simply recapitulation of results with no discussion of the published literature in the context of the current work. The discussion really needs to be entirely rewritten, or the authors need to write a combined results in discussion section if allowed by the journal format.

As noted above, the discussion has been revised and shortened to improve clarity and quality.

Page 15:
Content: "The minimum antimicrobial concentration of NH3 was determined in this study by inoculating ST into CHES buffer, adjusted to pH 9, with varying levels of ammonium sulfate added to achieve 0.04, 0.09, 0.18, 0.26, and 0.35 M NH3 (Table 3). The minimum antimicrobial concentration of NH3 was 0.04 M, which led to a 7.4 log CFU/mL reduction of ST within 24 h (Fig. 2B). "
Comment: These sentences are methods and results, and should be deleted from the discussion.

These statements have been removed or otherwise revised to improve the discussion.

Page 16:
Content: "The present study examined the mechanisms of NH3 production and the interactions between urea, urease, CU, and the ST inoculum. The urea (1% and 2%), urease, and CU control treatments (pH 9) resulted in Salmonella reductions of 3.4-4.6 log CFU/mL, whereas CHES (pH 9) control resulted in a 4.25-log reduction after 24 h (Fig. 3A-D and 2A)."
Comment: Unfortunately the only sentence in this paragraph which is really discussion is the first two sentences. From the sentence noted above onward to the end of the paragraph is simply a recapitulation of results. The authors make no attempt to discuss their findings in the context of the rest of the literature. This paragraph needs to be rewritten or deleted.

These statements have been removed or otherwise revised to improve the discussion.

Page 17:
Content: "Modeling was used in this study to describe the inactivation kinetics and assess the microbial stress response of S. Typhimurium to NH3 in solution."
Comment: The paragraph that starts with this sentence is also simply a recapitulation of the results. There is essentially no discussion, therefore this paragraph should be deleted.

This paragraph has been significantly re-worked to improve the discussion.

Page 18:
Content: "Except for the 0.04 M NH3 treatment, all NH3 and NH3-producing treatments were best fit to the Weibull model."
Comment: This paragraph sites no references, therefore it is not discussion, it is once again a recapitulation of the results and should be deleted.

This paragraph has been revised.

Page 18:
Content: "Among the NH3 concentration treatments, the δ and p values decreased as the concentration of NH3 was increased."
Comment: This paragraph is almost entirely a recapitulation of results with very little discussion. References are cited in the final sentence, but very little attempt made to discuss them in the context of the current work.

This paragraph has been revised.

Page 27:
Content: "Experiment "
Comment: This does not seem like the right header for this column in the table. Maybe experimental variable?

This heading in Table 1 has been adjusted as suggested.

Page 33:
Content: "Figure 1. Survival of Salmonella Typhimurium in buffer solutions (PBS, CHES) at various pH levels (5, 7,"
Comment: The results shown in the figure for TAN pH 9 are misleading. If no colonies were recovered at the limit of detection, they should not be plotted on the graph.

The limit of detection line in all figures has been corrected to prevent this issue.

Page 34:
Content: "Figure 2."
Comment: In this figure and those following, the authors need to be very careful about reporting results at the limit of detection. If they did not detect any colonies it is misleading to report zero log CFU. This can have profound effects on the model fitting, especially since data were only collected at three time points. If the last time point no colonies were detected, then two points determine a straight line. If the authors had chosen a different final sampling time and also found no colonies, this could change the model significantly.

The limit of detection line in all figures has been corrected to prevent this issue.

Reviewer #2 (Comments for the Author):

The authors of this study suggest that ammonia in poultry litter may affect the survival of Salmonella. This is an important topic and over all the manuscript is informative. The breakdown of urea to ammonia or other products can occur in various ways in the environment. Some confusion in the manuscript relates to this complex system. Overall, the results and discussion are clear, but the introduction is less clear. Perhaps the experiment can be set up and better described in the introduction.

The application and direction of study is unclear. Are the authors describing ammonia as if it would be a result or aspect of amended soils? Or are the authors describing ammonia gas produced during poultry production, in which case the age of the birds and the history of the house clean out are critical points of information. If this is a more basic study, as the title may suggest, then this should be clarified. The introduction seems to contain relevant information from all areas.
The story of ammonia and urea in soils is complicated, and the gaseous ammonia story can also be complicated, so the authors should be clear in terms of the narrative being written with the introduction.

The authors appreciate the comments and feedback on the manuscript. Lines 61-62, at the end of the first introduction paragraph have been added to clarify that this study is meant as an investigation of ammonia as an important factor in the survival of *Salmonella* in poultry litter, albeit outside of the litter environment. The authors are clear to avoid discussing manure-amended soil and ammonia in soil. This manuscript is solely concerned with *Salmonella* and ammonia in poultry litter.

Line 41-42 of the Importance could be reworded, as a sentence like "Previous studies..." should have references to support it, but references are not typically included in the Importance section.

These lines (40-41) have been revised to omit specific references to previous studies.

Lines 63-78 include an excellent discussion about the breakdown of ammonia. It should be made clear that ammonia transmission and breakdown in the soil is different from gaseous ammonia. It is not clear if this is true or not based on the writings here. Likewise in lines 84-85 the authors are discussing gaseous ammonia being applied to poultry houses.
There should be a discussion of breakdown of ammonia and ammonium salts in the soil.

The authors are careful to only mention the word soil in terms of biological soil amendments and avoid any discussion of manure-amended soils. The manuscript is focused on ammonia as a potential factor that may affect the survival of *Salmonella* in poultry litter. Discussions of ammonia in soil are outside of the scope of this work.

The authors should be clear about the spelling and writing of the word "unionized".

This has been corrected to a consistent spelling of the word “un-ionized”.

December 20, 2021

Prof. Keith Schneider
University of Florida
Food Science and Human Nutrition
359 FSHN Bldg.
Newell Dr.
Gainesville, FL 32611

Re: Spectrum01850-21R1 (Antimicrobial efficacy of un-ionized ammonia (NH_3) against Salmonella Typhimurium in buffered solutions with variable pH, NH_3 concentrations, and urease-producing bacteria)

Dear Prof. Keith Schneider:

Thank you for submitting your manuscript to Microbiology Spectrum. As you will see your paper is very close to acceptance. Please modify the manuscript along the lines I have recommended. As these revisions are quite minor, I expect that you should be able to turn in the revised paper in less than 30 days, if not sooner. If your manuscript was reviewed, you will find the reviewers' comments below.

When submitting the revised version of your paper, please provide (1) point-by-point responses to the issues I raised in your cover letter, and (2) a PDF file that indicates the changes from the original submission (by highlighting or underlining the changes) as file type "Marked Up Manuscript - For Review Only". Please use this link to submit your revised manuscript. Detailed instructions on submitting your revised paper are below.

Link Not Available

Thank you for the privilege of reviewing your work. Below you will find instructions from the Microbiology Spectrum editorial office and comments generated during the review.

The ASM Journals program strives for constant improvement in our submission and publication process. Please tell us how we can improve your experience by taking this quick Author Survey.

Sincerely,

Sandeep Tamber

Editor, Microbiology Spectrum

Comments:

need to clarify log transformation of the LOD:

(lines 172 173) "When ST populations fell below the LOD (1 CFU/mL), samples were assigned a value of 0 CFU/mL. This resulted in a log-transformed LOD of 0.30 log CFU/mL"

Should it read - samples were assigned a value of 1, therefore 1+1 = 2 = log 0.3?

Preparing Revision Guidelines
To submit your modified manuscript, log onto the eJP submission site at https://spectrum.msubmit.net/cgi-bin/main.plex. Go to Author Tasks and click the appropriate manuscript title to begin the revision process. The information that you entered when you first submitted the paper will be displayed. Please update the information as necessary. Here are a few examples of required updates that authors must address:

- point-by-point responses to the issues I raised in your cover letter
- Upload a compare copy of the manuscript (without figures) as a "Marked-Up Manuscript" file.

• Each figure must be uploaded as a separate file, and any multipanel figures must be assembled into one file.
• Manuscript: A .DOC version of the revised manuscript
• Figures: Editable, high-resolution, individual figure files are required at revision, TIFF or EPS files are preferred

For complete guidelines on revision requirements, please see the journal Submission and Review Process requirements at https://journals.asm.org/journal/Spectrum/submission-review-process. **Submissions of a paper that does not conform to Microbiology Spectrum guidelines will delay acceptance of your manuscript.** "

Please return the manuscript within 60 days; if you cannot complete the modification within this time period, please contact me. If you do not wish to modify the manuscript and prefer to submit it to another journal, please notify me of your decision immediately so that the manuscript may be formally withdrawn from consideration by Microbiology Spectrum.

If your manuscript is accepted for publication, you will be contacted separately about payment when the proofs are issued; please follow the instructions in that e-mail. Arrangements for payment must be made before your article is published. For a complete list of **Publication Fees**, including supplemental material costs, please visit ourwebsite.

Corresponding authors may join or renew ASM membership to obtain discounts on publication fees. Need to upgrade your membership level? Please contact Customer Service at Service@asmusa.org.

Thank you for submitting your paper to Microbiology Spectrum.

Reviewer comments:

Reviewer #1 (Comments for the Author):

The authors have performed a nice study investigating the behavior of salmonella in solution looking at the effect of ammonia and pH on rate of an activation. This research may inform future applied research on salmonella inactivation in poultry litter.

The work is generally sound, and my comments are generally minor.

There are some issues with reporting samples below the detection limit with being at the detection limit which might have implications for modeling or other conclusions.

I would also note that much of the discussion does not really discuss the published literature and needs to be significantly rewritten or combined with results if that is allowed by the journal.

The authors appreciate the reviewers' detailed comments and the opportunity to improve the manuscript for further consideration. The limit of detection has been clarified in the methods section and all figures have been corrected to accurately show the limit of detection. In addition, the discussion has been significantly re-worked to improve its clarity and quality for the reader.

Page 2:
Content: "and production efficacy of NH3."
Comment: Unclear what this phrase means.

This phrase has been revised to improve clarity.

Page 2:
Content: "Salmonella was completely inactivated (~ 8 log CFU/mL reduction)"
Comment: "Completely inactivated" depends upon starting concentration and detection limit. Better just to report the log reduction as the authors do here and delete this phrase throughout the document.

This term has been removed and replaced with the log reduction value.

Page 2:
Content: "while 0.04 M NH3 represented the minimum antimicrobial concentration tested (7.40 log CFU/mL reduction after 24 h). "
Comment: Awkward phrasing. Maybe divide the sentence into two?

This statement has been split into another sentence to improve clarity.

Page 2:
Content: "which completely inactivated Salmonella within 12 h."
Comment: This phrase is unscientific and not helpful. Please eliminate throughout the manuscript

This term has been removed and revised to remain within the abstract word limit.

Page 2:
Content: "completely inactivated"
Comment: Same comment

This term has been removed and revised to remain within the abstract word limit.

Page 3:
Content: "Poultry litter is recognized as an environmental source of Salmonella dissemination among birds in poultry houses (7-9). Poultry litter is a mixture of poultry excreta, feathers, wasted feed, and bedding materials from poultry houses (10)."
Comment: Better to reverse the order of these two sentences. Explain what poultry litter is before talking about why it's a problem.

These sentences have been reversed at the reviewer's suggestion to improve the readability of the manuscript.

Page 4:
Content: "S. Typhimurium in poultry litter was reduced by 10 log CFU/mL"
Comment: First, it seems impossible that a log reduction this high could be calculated. Second, poultry litter is measured in grams not mL. Please check the reference and confirm.

Turnbull and Snoeyenbos (1973) specify that their inoculum was 10^{10} cells/mL. They used standard plating methods to recover *Salmonella* from the litter and all figures use the units of log cells/mL. Certainly, in a modern context, this high inoculum level may seem unusual. Therefore, the authors have chosen to adjust this reference in a way that remains factual, yet less peculiar to modern readers.

Page 9:
Content: "Bacterial plate counts (CFU/mL) were log-transformed [log10 (CFU/mL + 1)] for statistical analysis. When ST populations fell below the LOD (1 CFU/mL; 0 log CFU/mL), samples were assigned a value of 0 CFU/mL."
Comment: This doesn't make sense and it may result in incorrect models.

This statement has been revised to correct the explanation of the log-transformed LOD and negative samples. Figures have also been revised to the correct LOD of 0.30 log CFU/mL [$\log_{10}$ (1 + 1)] according to the method described. This method was used to avoid the use of a negative log scale for censored values of negative samples.

Page 11:
Content: "The calculated un-ionized ammonia"
Comment: The word un-ionized is also spelled elsewhere in the manuscript without a hyphen. I think the authors should be consistent, and I prefer the hyphenated form.

This has been corrected to a consistent spelling of "un-ionized".

Page 14:
Content: "DISCUSSION"
Comment: As noted below, a great deal of the discussion is simply recapitulation of results with no discussion of the published literature in the context of the current work. The discussion really needs to be entirely rewritten, or the authors need to write a combined results in discussion section if allowed by the journal format.

As noted above, the discussion has been revised and shortened to improve clarity and quality.

Page 15:
Content: "The minimum antimicrobial concentration of NH3 was determined in this study by inoculating ST into CHES buffer, adjusted to pH 9, with varying levels of ammonium sulfate added to achieve 0.04, 0.09, 0.18, 0.26, and 0.35 M NH3 (Table 3). The minimum antimicrobial concentration of NH3 was 0.04 M, which led to a 7.4 log CFU/mL reduction of ST within 24 h (Fig. 2B). "
Comment: These sentences are methods and results, and should be deleted from the discussion.

These statements have been removed or otherwise revised to improve the discussion.

Page 16:
Content: "The present study examined the mechanisms of NH3 production and the interactions between urea, urease, CU, and the ST inoculum. The urea (1% and 2%), urease, and CU control treatments (pH 9) resulted in Salmonella reductions of 3.4-4.6 log CFU/mL, whereas CHES (pH 9) control resulted in a 4.25-log reduction after 24 h (Fig. 3A-D and 2A)."
Comment: Unfortunately the only sentence in this paragraph which is really discussion is the first two sentences. From the sentence noted above onward to the end of the paragraph is simply a recapitulation of results. The authors make no attempt to discuss their findings in the context of the rest of the literature. This paragraph needs to be rewritten or deleted.

These statements have been removed or otherwise revised to improve the discussion.

Page 17:
Content: "Modeling was used in this study to describe the inactivation kinetics and assess the microbial stress response of S. Typhimurium to NH3 in solution."
Comment: The paragraph that starts with this sentence is also simply a recapitulation of the results. There is essentially no discussion, therefore this paragraph should be deleted.

This paragraph has been significantly re-worked to improve the discussion.

Page 18:
Content: "Except for the 0.04 M NH3 treatment, all NH3 and NH3-producing treatments were best fit to the Weibull model."
Comment: This paragraph sites no references, therefore it is not discussion, it is once again a recapitulation of the results and should be deleted.

This paragraph has been revised.

Page 18:
Content: "Among the NH3 concentration treatments, the δ and p values decreased as the concentration of NH3 was increased."
Comment: This paragraph is almost entirely a recapitulation of results with very little discussion. References are cited in the final sentence, but very little attempt made to discuss them in the context of the current work.

This paragraph has been revised.

Page 27:
Content: "Experiment "
Comment: This does not seem like the right header for this column in the table. Maybe experimental variable?

This heading in Table 1 has been adjusted as suggested.

Page 33:
Content: "Figure 1. Survival of Salmonella Typhimurium in buffer solutions (PBS, CHES) at various pH levels (5, 7,"
Comment: The results shown in the figure for TAN pH 9 are misleading. If no colonies were recovered at the limit of detection, they should not be plotted on the graph.

The limit of detection line in all figures has been corrected to prevent this issue.

Page 34:
Content: "Figure 2."
Comment: In this figure and those following, the authors need to be very careful about reporting results at the limit of detection. If they did not detect any colonies it is misleading to report zero log CFU. This can have profound effects on the model fitting, especially since data were only collected at three time points. If the last time point no colonies were detected, then two points determine a straight line. If the authors had chosen a different final sampling time and also found no colonies, this could change the model significantly.

The limit of detection line in all figures has been corrected to prevent this issue.

Reviewer #2 (Comments for the Author):

The authors of this study suggest that ammonia in poultry litter may affect the survival of Salmonella. This is an important topic and over all the manuscript is informative. The breakdown of urea to ammonia or other products can occur in various ways in the environment. Some confusion in the manuscript relates to this complex system. Overall, the results and discussion are clear, but the introduction is less clear. Perhaps the experiment can be set up and better described in the introduction.

The application and direction of study is unclear. Are the authors describing ammonia as if it would be a result or aspect of amended soils? Or are the authors describing ammonia gas produced during poultry production, in which case the age of the birds and the history of the house clean out are critical points of information. If this is a more basic study, as the title may suggest, then this should be clarified. The introduction seems to contain relevant information from all areas.
The story of ammonia and urea in soils is complicated, and the gaseous ammonia story can also be complicated, so the authors should be clear in terms of the narrative being written with the introduction.

The authors appreciate the comments and feedback on the manuscript. Lines 61-62, at the end of the first introduction paragraph have been added to clarify that this study is meant as an investigation of ammonia as an important factor in the survival of *Salmonella* in poultry litter, albeit outside of the litter environment. The authors are clear to avoid discussing manure-amended soil and ammonia in soil. This manuscript is solely concerned with *Salmonella* and ammonia in poultry litter.

Line 41-42 of the Importance could be reworded, as a sentence like "Previous studies..." should have references to support it, but references are not typically included in the Importance section.

These lines (40-41) have been revised to omit specific references to previous studies.

Lines 63-78 include an excellent discussion about the breakdown of ammonia. It should be made clear that ammonia transmission and breakdown in the soil is different from gaseous ammonia. It is not clear if this is true or not based on the writings here. Likewise in lines 84-85 the authors are discussing gaseous ammonia being applied to poultry houses.
There should be a discussion of breakdown of ammonia and ammonium salts in the soil.

The authors are careful to only mention the word soil in terms of biological soil amendments and avoid any discussion of manure-amended soils. The manuscript is focused on ammonia as a potential factor that may affect the survival of *Salmonella* in poultry litter. Discussions of ammonia in soil are outside of the scope of this work.

The authors should be clear about the spelling and writing of the word "unionized".

This has been corrected to a consistent spelling of the word “un-ionized”.

December 22, 2021

Prof. Keith Schneider
University of Florida
Food Science and Human Nutrition
359 FSHN Bldg.
Newell Dr.
Gainesville, FL 32611

Re: Spectrum01850-21R2 (Antimicrobial efficacy of un-ionized ammonia (NH_3) against Salmonella Typhimurium in buffered solutions with variable pH, NH_3 concentrations, and urease-producing bacteria)

Dear Prof. Keith Schneider:

Your manuscript has been accepted, and I am forwarding it to the ASM Journals Department for publication. You will be notified when your proofs are ready to be viewed.

The ASM Journals program strives for constant improvement in our submission and publication process. Please tell us how we can improve your experience by taking this quick Author Survey.

As an open-access publication, Spectrum receives no financial support from paid subscriptions and depends on authors' prompt payment of publication fees as soon as their articles are accepted. You will be contacted separately about payment when the proofs are issued; please follow the instructions in that e-mail. Arrangements for payment must be made before your article is published. For a complete list of **Publication Fees**, including supplemental material costs, please visit ourwebsite.

Corresponding authors may join or renew ASM membership to obtain discounts on publication fees. Need to upgrade your membership level? Please contact Customer Service at Service@asmusa.org.

Thank you for submitting your paper to Spectrum.

Sincerely,

Sandeep Tamber
Editor, Microbiology Spectrum

Journals Department
American Society for Microbiology
1752 N St., NW
Washington, DC 20036
E-mail: spectrum@asmusa.org

Supplemental Tables: Accept
